# Ultrasonic vocalizations in house mice depend upon genetic relatedness of mating partners and correlate with subsequent reproductive success

**DOI:** 10.1186/s12983-020-00353-1

**Published:** 2020-04-03

**Authors:** Doris Nicolakis, Maria Adelaide Marconi, Sarah M. Zala, Dustin J. Penn

**Affiliations:** Konrad Lorenz Institute of Ethology, Department of Interdisciplinary Life Sciences, University of Veterinary Medicine, Vienna, Savoyenstraße 1a, 1160 Vienna, Austria

**Keywords:** *Mus musculus musculus*, House mice, Wild derived, USV, Ultrasonic vocalizations, Reproductive success, Genetic relatedness, Adaptive functions

## Abstract

**Background:**

Courtship vocalizations are used by males of many species to attract and influence the behavior of potential mating partners. Our aim here was to investigate the modulation and reproductive consequences of courtship ultrasonic vocalizations (USVs) in wild-derived house mice (*Mus musculus musculus*). The courtship USVs of male mice are surprisingly complex and are composed of a variety of different syllable types. Our specific aims were to test whether (1) the emission of courtship USVs depends upon the kinship of a potential mating partner, and (2) whether USV emission during courtship affects the pairs’ subsequent reproductive success.

**Results:**

We experimentally presented males with an unfamiliar female that was either genetically related or unrelated, and we recorded USV emission, first while the sexes were separated by a perforated partition and then during direct interactions, after removing the partition. USVs were detected by the Automatic Mouse Ultrasound Detector (A-MUD) and manually classified into 15 syllable types. The mice were kept together to test whether and how courtship vocalizations predict their subsequent reproductive success. We found that the mice significantly increased their amount of vocalizations (vocal performance) and number of syllable types (vocal repertoire) after the partition was removed and they began interacting directly. We show that unrelated pairs emitted longer and more complex USVs compared to related pairs during direct interactions. Unrelated pairs also had a greater reproductive success compared to related pairs, and in addition we found a negative correlation between the mean length and amount of vocalizations with the latency to their first litter.

**Conclusion:**

Our study provides evidence that house mice modulate the emission of courtship USVs depending upon the kinship of potential mating partners, and that courtship USVs correlate with reproductive success.

## Background

Courtship vocalizations are produced in many species, usually by males, as a mechanism to attract and influence the behavior of potential mates [1, 2]. In some birds, exposure to male courtship song can induce ovarian development in females [3] and copulation solicitation behaviors [4]. Courtship vocalizations can reveal a surprising amount of information about a male to potential mates, including their fertility, genetic quality and species or individual identity [reviewed in 1]. Many studies on sexual selection in mammals, however, have focused on vocalizations emitted during competitive male-male interactions, and though there is increasing evidence for female-choice [5], these studies have mainly investigated chemical signals (olfactory communication). Surprisingly little is known about whether and how mammalian vocalizations evolve through female choice. Here, we investigated the functions of the courtship ultrasonic vocalizations (USVs) of wild-derived male house mice (*Mus musculus musculus).*

The USVs of male house mice are complex and have features similar to birdsong [6]. Males emit USVs mainly during courtship and mating, however, their functions are still unclear (reviewed in [7–10]). The vast majority of studies on mouse USVs have been conducted on inbred laboratory strains (*Mus laboratorius)*, and USVs are often used as a tool to investigate neurodevelopmental and speech disorders [11, 12]. Previous studies suggest that USVs provide a reliable signal of male sexual arousal or motivation (reviewed in [13]). The complexity of male USVs is increased during the course of courtship and particularly just before copulation [14] and mice of both sexes emit vocalizations at a higher rate and higher frequencies during opposite- compared to same-sex interactions [15]. Female mice show approach behavior towards playbacks of male vocalizations [16–18], and they show preferences for USVs with more frequency jumps [19]. Several hypotheses have been proposed to explain why females are attracted to male USVs (reviewed in [7]), and so far playback studies provide evidence for two potential functions: (1) species recognition, as *Mus musculus* females are more attracted to playbacks of male USV of their own species compared to those of *Mus spicilegus* [20]; and (2) kin recognition, as females are more attracted to the USVs of non-kin compared to those of their siblings [17]. In this study we aimed to test whether males modulate their USV emission depending upon the genetic compatibility of a potential mating partner (kin recognition), and we tested whether courtship vocalizations predict a mating pairs’ subsequent reproductive success.

It has long been suggested that the courtship USVs of male mice influence mating and reproductive success, and yet only two studies have tested this hypothesis to our knowledge. First, Asaba et al. [18] recorded vocalizations during interactions of males with a female after being housed with a different female for 4 months. They found a correlation between the number of deliveries during this time and the number of USVs males emitted when they were later recorded with the other female. However, it is unclear whether male USV emission influenced male mating success, or vice versa. Second, Kanno and Kikusui [21] recorded males with a novel virgin female both before and after they were housed with another female for 2 weeks. They compared males that emitted USVs with males that did not vocalize before or after co-housing, and they found that vocalizing males sired more offspring than non-vocalizing males during the co-housing phase. This study recorded males before and after housing with a female, but only compared vocalizing versus non-vocalizing males. Thus, it is still unknown whether any other variation in male USVs predicts reproductive success. Also, these studies were both conducted on laboratory mice (C57BL/6 J), which are very different from wild mice (laboratory strains are selected for rapid reproduction, and differ in their vocalizations, courtship and mating behavior), and therefore, we aimed to investigate the adaptive functions of courtship USVs in wild-derived house mice.

In the present study we experimentally manipulated the genetic compatibility (relatedness) of breeding pairs by presenting males with an unfamiliar female, which was either genetically related or not (no-choice mate preference). We recorded the USVs emitted both, before and after removing a perforated divider, and we then tested whether the mice modulate the emission of courtship USVs depending on their genetic relatedness. This experiment allowed us to test whether males alter their USV emission during the early phases of courtship and also to test whether males are able to recognize and show preferences for unrelated over genetically related potential mating partners. We expected that if the mice show kin recognition and inbreeding avoidance, then they will emit more USVs and a more complex repertoire when paired with unrelated females. Finally, we tested whether male courtship USV emissions influence the pairs’ subsequent reproductive success.

## Results

### Phases of courtship

**Fig. 1:**
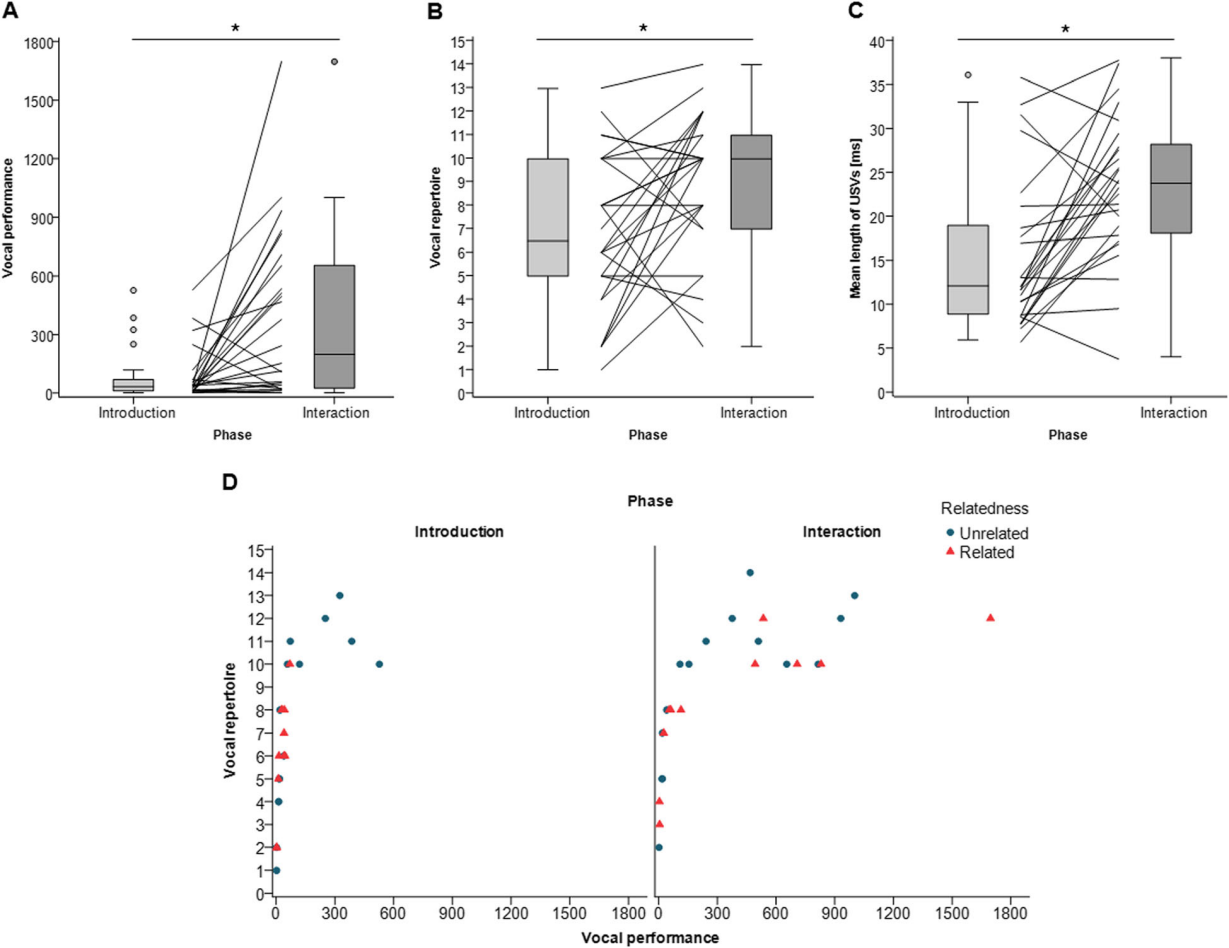
USV emission during the introduction phase compared to the interaction phase. Boxplots show median (center line), interquartile range (box), 95% variation (whiskers) and outliers (circles) of (**a**) the vocal performance (total number of USVs emitted during 10 min), (**b**) the vocal repertoire (number of syllable types emitted during 10 min) and (**c**) the mean length of USVs (ms). **d** Relationship between the total number of USVs (vocal performance) and number of syllable types (vocal repertoire) emitted by unrelated (blue circles) and related (red triangles) pairs during the introduction (left) and interaction phase (right). * *p* < 0.05

We first investigated whether and how the males modulated their USV emission when they were presented with a female, first while separated by a clear, perforated divider (*introduction phase*) and then during direct contact, after the divider was removed (*interaction phase*). Overall, the mice emitted 5x more USVs and produced more types of syllables during than before direct interactions (vocal performance: Wilcoxon test: *n* = 26, Z = − 3.264, *p* = 0.001, Fig. 1a; vocal repertoire: Wilcoxon test: *n* = 26, Z = − 3.912, *p* < 0.001, Fig. 1b; Additional file 1: Table S1). In both phases there was a positive correlation between vocal performance and vocal repertoire, so that the mice that emitted more USVs also emitted more syllable types (Spearman correlation: introduction: *n* = 26, *r*_s_ = 0.926, *p* < 0.001, interaction: *n* = 26, *r*_s_ = 0.852, *p* < 0.001) (Fig. 1d). The vocal repertoire first increased with the number of vocalizations but then plateaued after circa 10 syllable types. Hence, the relationship between vocal performance and repertoire follows a logarithmic curve. During direct interactions the mice also emitted longer syllables compared to the introduction phase (Wilcoxon test: *n* = 26, Z = − 3.467, *p* = 0.001, Additional file 1: Table S1) (Fig. 1c). Therefore, we examined these two phases separately for our subsequent analyses.

### Female sexual receptivity

**Fig. 2:**
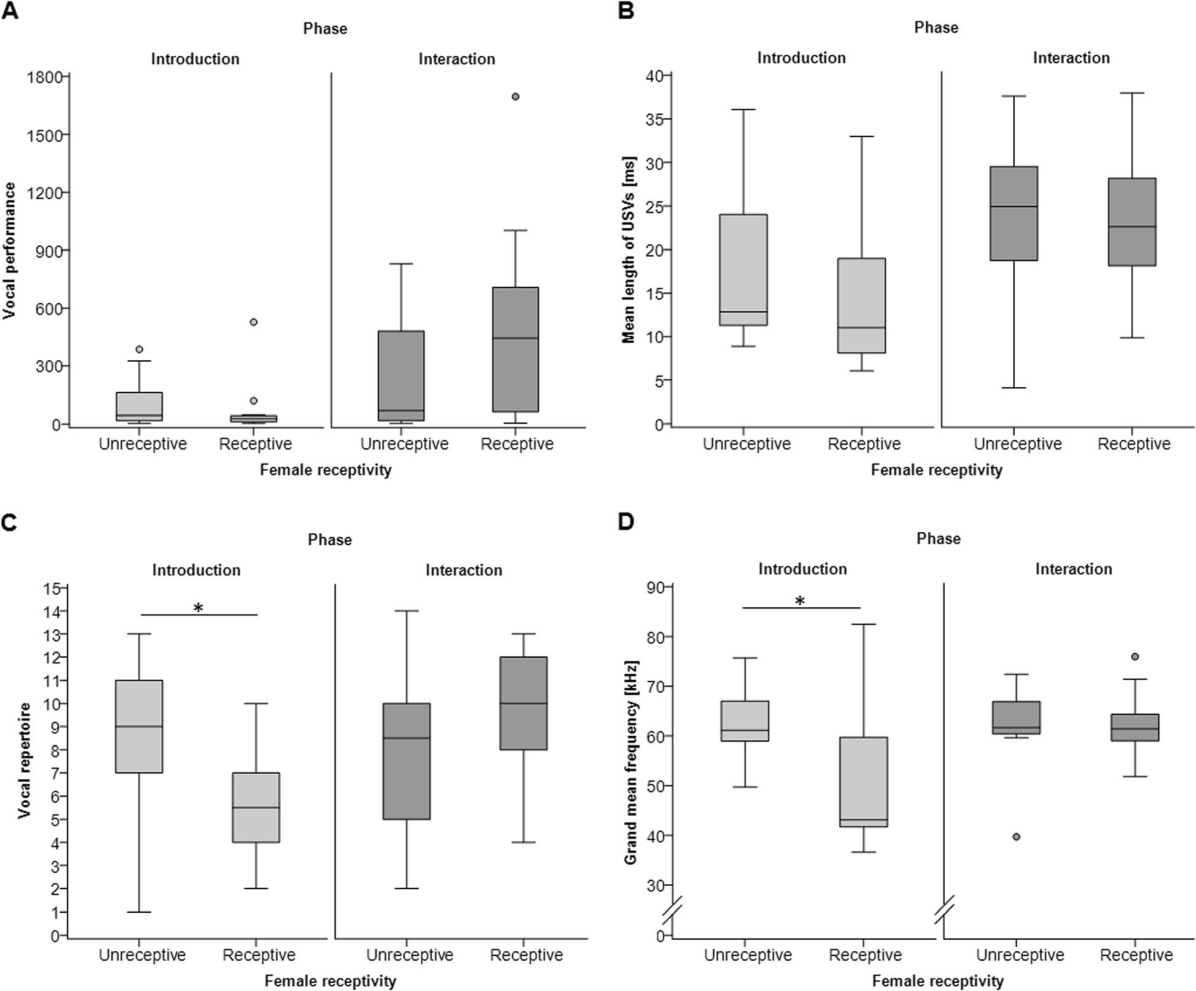
USV emission during the introduction versus interaction phase when females were unreceptive or receptive. Boxplots show median (center line), interquartile range (box), 95% variation (whiskers) and outliers (circles) of (**a**) the vocal performance (total number of USVs emitted during 10 min), (**b**) the mean length of USVs (ms) (**c**) the vocal repertoire (number of syllable types emitted during 10 min) and (**d**) the grand mean frequency of USVs (kHz). * *p* < 0.05

We examined whether USV emission was influenced by female estrous state. During the introduction phase, vocal performance did not differ when males were exposed to females of any of the four estrous states (Kruskal-Wallis test: *n* = 26, χ^2^ = 3.169, *p* = 0.366). Visual inspection suggests that during the interaction phase more USVs were emitted when females were in proestrus than other stages; however, there was also no significant difference among the four estrous states (Kruskal-Wallis test: *n* = 26, χ^2^ = 5.469, *p* = 0.141). Next, we combined females in proestrus and estrus into “receptive females” and females in metestrus and diestrus into “unreceptive females.” Males had a higher median vocal performance during direct interactions with a receptive female compared to unreceptive females, however, this difference was not statistically significant (Mann-Whitney U test: *n* = 26, introduction: Z = − 1.313, *p* = 0.189, interaction: *n* = 26, Z = − 1.698, *p* = 0.090, Additional file 1: Table S2) (Fig. 2a). Female receptivity had no significant effect on the mean length of USVs during either phase (Mann-Whitney U test: introduction: *n* = 26, Z = − 1.234, *p* = 0.217, interaction: *n* = 26, Z = − 0.309, *p* = 0.758, Additional file 1: Table S2) (Fig. 2b). Mice produced a larger vocal repertoire when presented with an unreceptive female (vs. a receptive female) during the introduction phase (Mann-Whitney U test: *n* = 26, Z = − 2.434, *p* = 0.015), but not during the direct interactions (Mann-Whitney U test: *n* = 26, Z = − 1.643, *p* = 0.100) (Fig. 2c, Additional file 1: Table S2). Female receptivity also influenced the grand mean frequency of USVs emitted during introduction (Mann-Whitney U test: *n* = 25, Z = − 2.502, *p* = 0.012) but not during direct interactions (Mann-Whitney U test: *n* = 26, Z = − 0.463, *p* = 0.643) (Fig. 2d, Additional file 1: Table S2) such that USVs emitted in the presence of receptive females had a lower grand mean frequency (50.67 ± 13.42 kHz) compared to unreceptive females (62.42 ± 7.73 kHz).

### Genetic relatedness

**Fig. 3:**
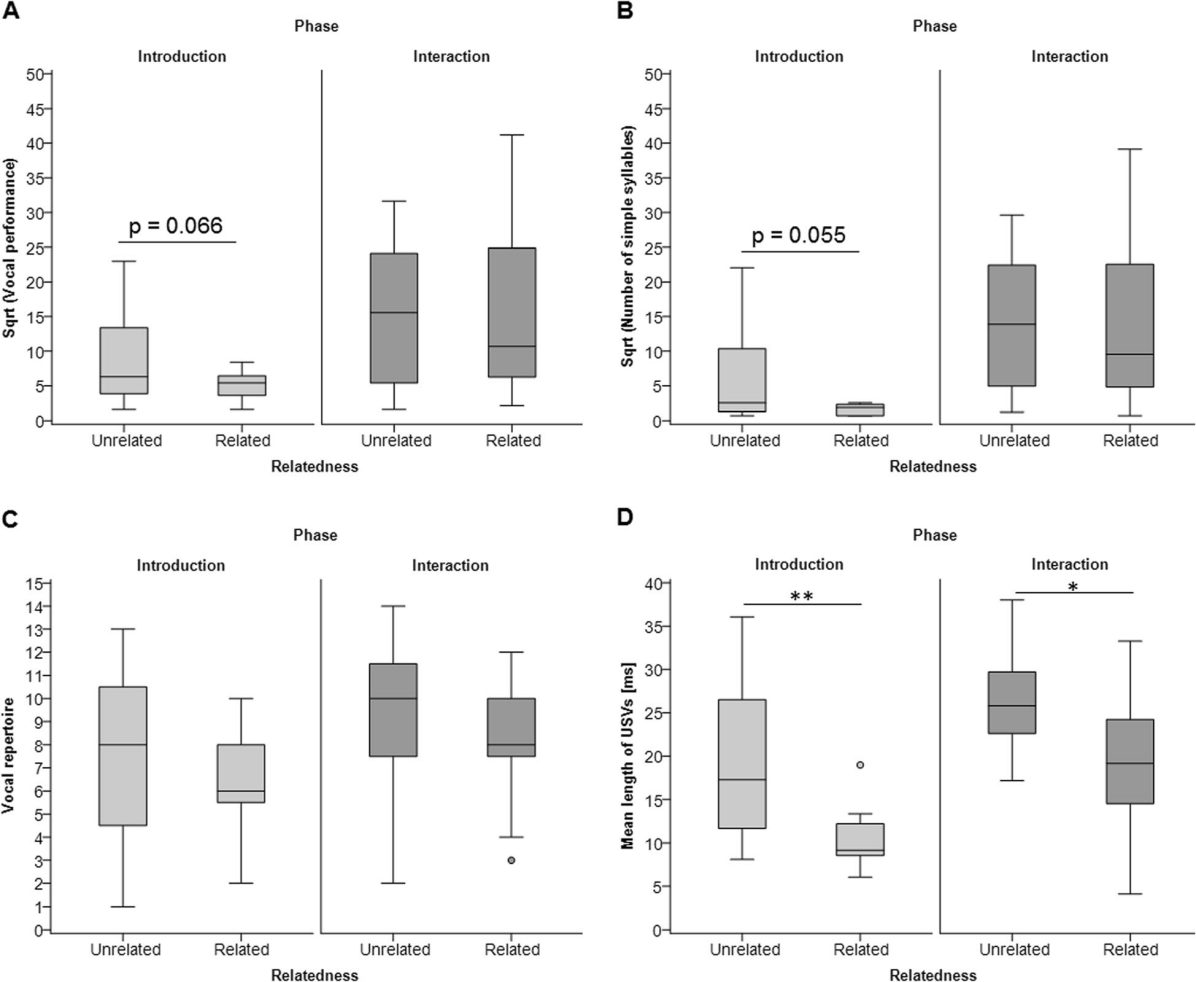
USV emission of unrelated and related pairs during the introduction versus interaction phase. Boxplots show median (center line), interquartile range (box), 95% variation (whiskers) and outliers (circles) of the (**a**) vocal performance (square-root-transformed total number of USVs emitted during 10 min) (**b**) number of simple syllables (square-root-transformed total number of simple syllables emitted during 10 min), (**c**) vocal repertoire (number of syllable types emitted during 10 min) and (**d**) mean length of USVs (ms). * *p* < 0.05, ** *p* < 0.01

We next tested whether USVs were modulated by presentation of a genetically related or unrelated partner. During the introduction phase, males tended to have a higher vocal performance when presented with an unrelated female compared to a related female (Welch’s t-test: *n* = 26, t = 1.963, *p* = 0.066), though not during direct interactions (t-test: *n* = 26, t = − 0.038, *p* = 0.9) (Fig. 3a, Additional file 1: Table S3). This trend was mainly due to males emitting more simple syllables when presented with unrelated compared to related females in the introduction phase (introduction: Mann-Whitney U test: *n* = 26, Z = − 1.917, *p* = 0.055**,** interaction: t-test: *n* = 26, t = − 0.005, *p* = 0.996) (Fig. 3b, see Additional file 1: Table S3). The vocal repertoire did not differ between unrelated and related pairs in any phase (introduction: Welch’s t-test: *n* = 26, t = 1.035, *p* = 0.311, interaction: t-test: *n* = 26, t = 0.773, *p* = 0.447) (Fig. 3c, Additional file 1: Table S3), however, unrelated mice always emitted longer USVs than related mice in both phases (introduction: Welch’s t-test: *n* = 26, t = 3.161, *p* = 0.005, interaction: t-test: *n* = 26, t = 2.449, *p* = 0.020) (Fig. 3d, Additional file 1: Table S3). These results were not influenced by female estrous state as there was no interaction between female receptivity and relatedness to the male (GZLM, interaction of receptivity*relatedness: vocal performance: introduction: *n* = 26, Wald-χ^2^ = 0.133, *p* = 0.715, interaction: *n* = 26, Wald-χ^2^ = 0.756, *p* = 0.388; vocal repertoire: introduction: *n* = 26, Wald-χ^2^ = 0.006, *p* = 0.937, interaction: *n* = 26, Wald-χ^2^ = 0.446, *p* = 0.504; mean USV length: introduction: *n* = 26, Wald-χ^2^ = 0.290, *p* = 0.590, interaction: *n* = 26, Wald-χ^2^ = 0.017, *p* = 0.896; Additional file 1: Table S4).

**Fig. 4:**
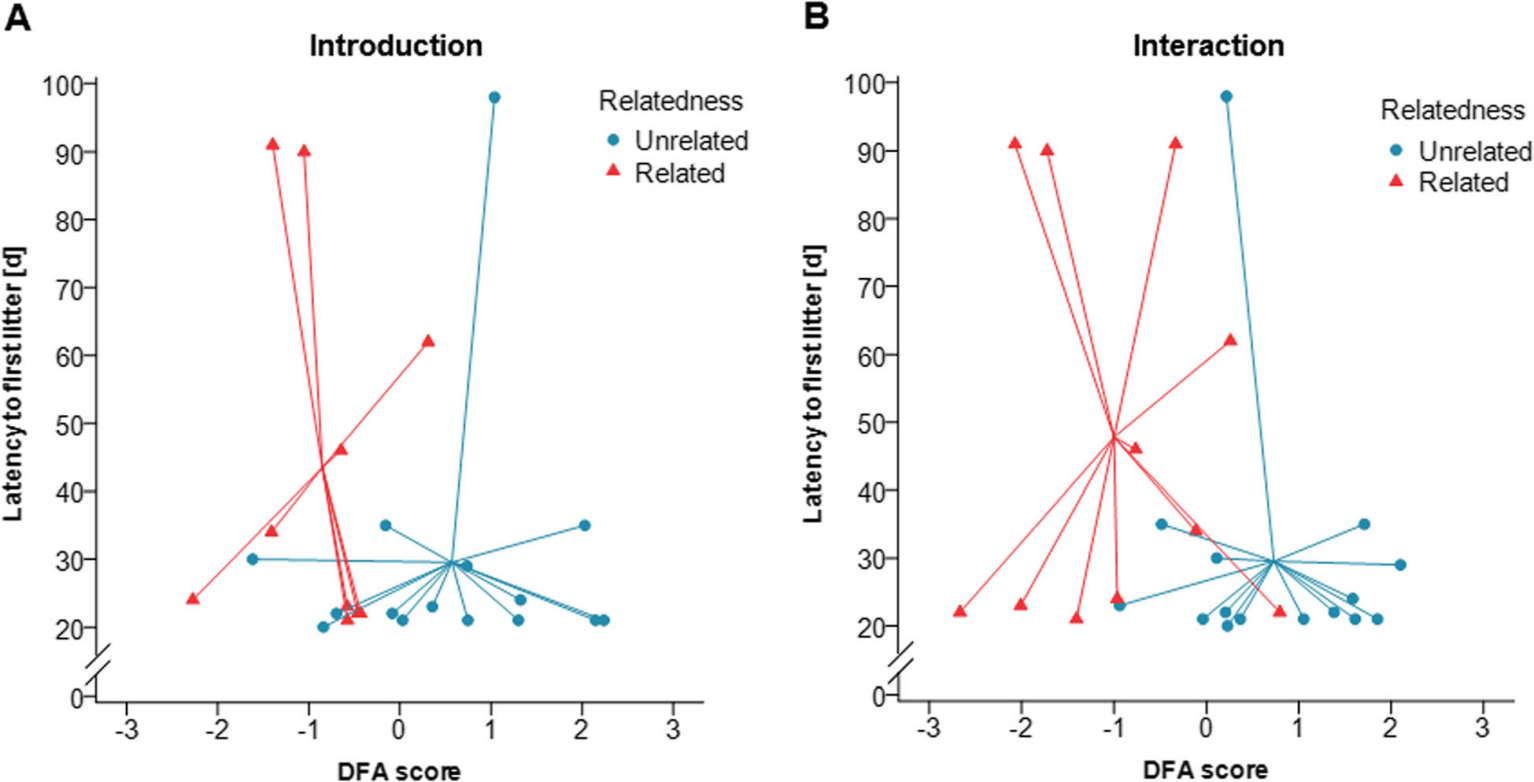
Discriminant function scores of USV emission and latency to reproduce. Each symbol represents one genetically unrelated (blue circles) or related (red triangles) breeding pair and lines connect individual pairs to the corresponding group centroid. **a** Introduction phase: larger DFA scores represent a higher number of simple syllables, longer durations and higher frequencies; smaller scores indicate a higher number of short syllables and lower frequencies. **b** Direct interaction phase: larger DFA scores represent longer USVs and a higher number of complex syllables; smaller scores indicate a higher number of short syllables.

We further investigated whether other features of USVs were influenced by a pair’s genetic relatedness running a discriminant function analysis (DFA) with the following parameters: mean USV length (ms), grand mean USV frequency (kHz), vocal repertoire, total number of short syllables (square-root-transformed), total number of simple syllables (square-root-transformed), and total number of complex syllables (square-root-transformed). These USV parameters tended to discriminate pairs of different relatedness during direct interactions (DFA: *n* = 26, Wilks’ Lambda = 0.558, canonical correlation = 0.665, *p* = 0.057), but not during introduction (DFA: *n* = 25, Wilks’ Lambda = 0.655, canonical correlation = 0.588, *p* = 0.206) (Fig. 4). Using cross-validation, the DFA was able to correctly classify 73% unrelated and 50% related pairs into the respective group in the introduction phase (overall: 64%, not cross-validated: 84%), and 66% unrelated and 63% related pairs in the interaction phase (overall 65.4%, not cross-validated: 80.8%). For each phase USV features could be combined into one discriminant function, which was plotted against the latency to the first litter (LFL) (Fig. 4) as a measure of reproductive success (see below). The parameters with the greatest discriminatory ability between related and unrelated pairs were number of short syllables, grand mean frequency and mean USV length in the introduction phase and number of simple syllables, mean USV length and number of short syllables in the interaction phase. Thus, in the introduction phase males emitted a larger number of simple syllables with a longer duration and higher frequency to unrelated females, whereas they emitted a larger number of short syllables at lower frequencies to related females (Fig. 4a). During direct interactions, unrelated mice emitted USVs with a longer duration and used a larger number of complex syllables, while related mice emitted a larger number of short and simple syllables (Fig. 4b).

**Fig. 5:**
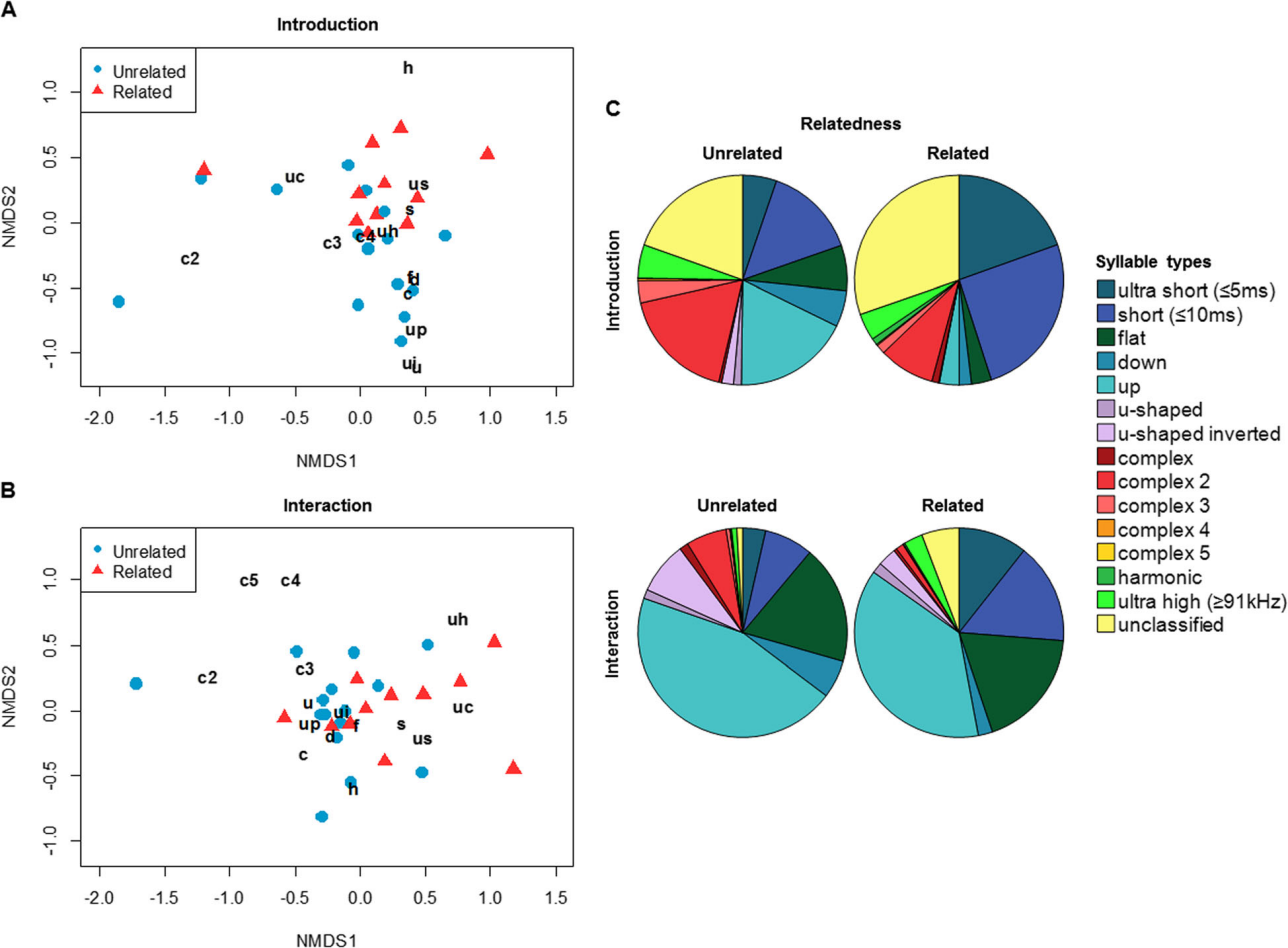
Syllable type usage presented in non-metric multidimensional scaling (nMDS) plots and pie charts. NMDS plots of syllable types emitted during (**a**) introduction and (**b**) interaction phase comparing unrelated pairs (blue dots) versus related pairs (red triangles). Letters in black indicate the syllable types; each symbol represents one breeding pair. Distances between the symbols represent similarities of breeding pairs in the syllable type usage. Short distances of symbols to letters indicate syllable types that were most representative for each breeding pair. **c** Pie charts representing proportions of each syllable type used by unrelated and related pairs during introduction and interaction phase. Both visualizations show similar results of syllable type usage

We then compared the different syllable types emitted by related versus unrelated pairs using multivariate analyses. The results showed that the number of syllables used per syllable type tended to differ between related and unrelated mice during the introduction phase (PERMANOVA: *n* = 26, F = 1.942, *p* = 0.062), but not during direct interactions (PERMANOVA: *n* = 26, F = 0.797, *p* = 0.481). Variances were larger in unrelated than related pairs during the introduction phase (permutation based analysis of multivariate group dispersions: *n* = 26, F = 4.314, *p* = 0.041). Since PERMANOVA assumes similar multivariate dispersions, these results should be treated with caution and interpreted only for exploratory purposes. In detail, 80% of the difference between the unrelated and related pairs during the introduction phase was explained by five syllable types (“up”, “uc”, “s”, “c2” and “us”) (Fig. 5a). Three syllable types (“up”, “c2” and “s”) showed a greater abundance when males were presented with an unrelated partner and two syllable types (“uc” and “us”) were emitted more often by related pairs (Fig. 5a, c). Visual inspection of the pie charts suggests that related mice emitted more ultrashort, short and unclassified syllables (“us”, “s” and “uc”) in both phases, whereas unrelated mice emitted more “up”, “u”, “ui”, “c2”, “c3” and “c4” syllables in both phases (Fig. 5c). These results are also consistent with the previous DFA showing that related mice emitted larger number of ultrashort and short syllables, whereas unrelated mice emitted a larger number of simple syllables during the introduction phase and a larger number of complex syllables during interactions (Fig. 4). Visualization of syllable type usage in a 2-dimensional space using non-metric multidimensional scaling (nMDS) plots provides a good representation of the data during the introduction phase (stress = 0.109) (Fig. 5a) and an intermediate representation for the interaction phase (stress = 0.150) (Fig. 5b). Visual comparison of pairs consisting of siblings and cousins show a similar distribution in syllable type usage, however, we did not conduct a statistical comparison due to the low sample sizes within groups (see Additional file 1: Figure S1).

### Reproductive success

**Fig. 6:**
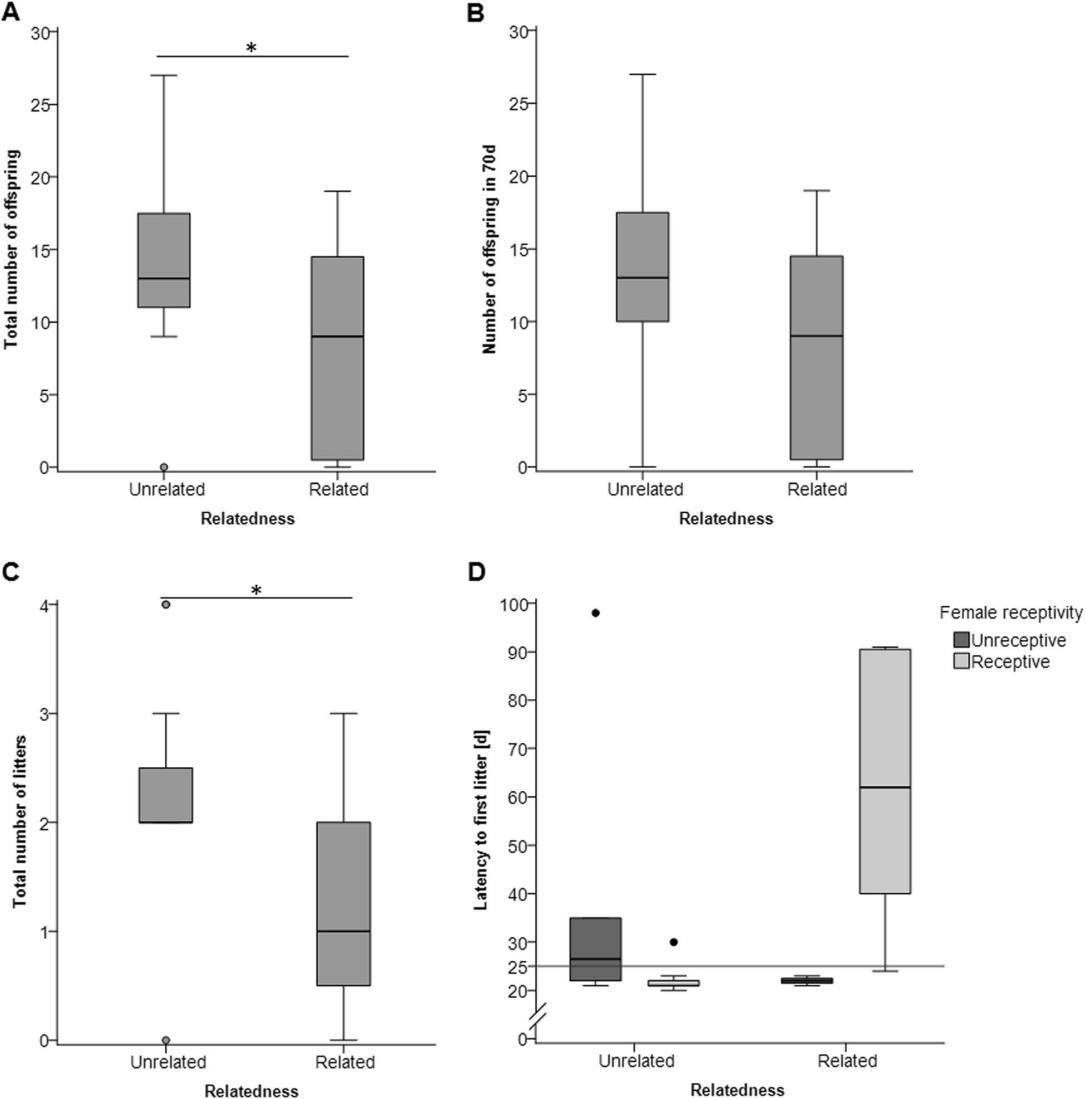
Reproductive success of unrelated and related breeding pairs. Boxplots show median (center line), interquartile range (box), 95% variation (whiskers) and outliers (circles) of (**a**) the total number of offspring during the entire breeding period, (**b**) the number of offspring born within 70d, and (**c**) the total number of litters during the entire breeding period. **d** Latency (days) to the first litter (LFL) comparing males paired with unrelated or related females, which were either sexually unreceptive (dark grey, *n* = 8 unrelated, 4 related) or receptive (light grey, *n* = 7 unrelated, 7 related) on the recording day. There was a significant interaction between the female receptivity and relatedness to the male. The line at 25d represents the division into pairs with a low LFL (<25d) vs. high LFL (>25d) (see later). * *p* < 0.05

We tested whether genetic relatedness influenced the reproductive success of the pairs, and found that unrelated pairs sired significantly more offspring than related pairs during the entire breeding period (t-test: *n* = 26, t = 2.215, *p* = 0.036) (Fig. 6a) When comparing the number of offspring born within 70d (i.e. the same time period for all breeding pairs), unrelated pairs still sired more offspring (13 ± 7) than related pairs (8 ± 7), however, this difference was not significant (t-test: *n* = 26, t = 1.783, *p* = 0.087) (Fig. 6b). Unrelated pairs gave birth to more litters (Mann-Whitney U test: *n* = 26, Z = − 2.381, *p* = 0.017) (Fig. 6c, Table 1), while the litter size did not significantly differ between unrelated and related pairs (t-test: *n* = 26, T = 1.344, *p* = 0.191; Table 1). Furthermore, unrelated pairs tended to have a shorter latency to the first litter (Mann-Whitney U test: *n* = 26, Z = − 1.832, *p* = 0.067) (Fig. 6d, Table 1) compared to related pairs, however this effect of relatedness depended on female receptivity. We found an interaction between the female’s receptivity and her relatedness to the male on the latency to the first litter (GZLM: *n* = 26, effect of relatedness: Wald-χ^2^ = 5.135, *p* = 0.023, effect of receptivity: Wald-χ^2^ = 5.530, *p* = 0.019, interaction relatedness*receptivity: Wald-χ^2^ = 24.391, *p* < 0.001). Among pairs with females that were initially receptive, unrelated pairs had a significantly shorter LFL than related pairs. When females were initially unreceptive, there was no difference in LFL between related and unrelated pairs (Fig. 6d).

**Table 1:** Statistical comparison of reproductive success (RS) between unrelated (*n* = 15) and related (*n* = 11) breeding pairs

		RS within entire breeding period		RS within 70 days	
**Mann-Whitney U test**		**Z**	***p*-value**	**Z**	***p*-value**
**Variable**	**Comparison**				
Latency to first litter	Unrelated vs. Related	−1.832	0.067	−1.913	0.056
Number of litters		−2.381	**0.017**	−2.096	**0.036**
**t-test**		**t**	***p*-value**	**t**	***p*-value**
**Variable**	**Comparison**				
Number of offspring	Unrelated vs. Related	2.215	**0.036**	1.783	0.087
N. offspring in first litter		1.356	0.188	1.356	0.188
N. offspring/litter		1.344	0.191	1.264	0.218
**GZLM**		**Wald-Chi-Square**	***p*-value**	**Wald-Chi-Square**	***p*-value**
**Variable**	**Effect**				
Log (Latency to first litter)	Relatedness	5.135	**0.023**	6.201	**0.013**
	Receptivity	5.530	**0.019**	6.744	**0.009**
	Relatedness*Receptivity	24.391	**< 0.001**	29.298	**< 0.001**

Results are shown for data including offspring delivered during the entire breeding period (85 ± 15d) and when using only offspring delivered within 70d. The second dataset represents equal breeding opportunities for all pairs. Results showing *p* < 0.05 are reported in bold

### USV emission and reproductive success

**Fig. 7:**
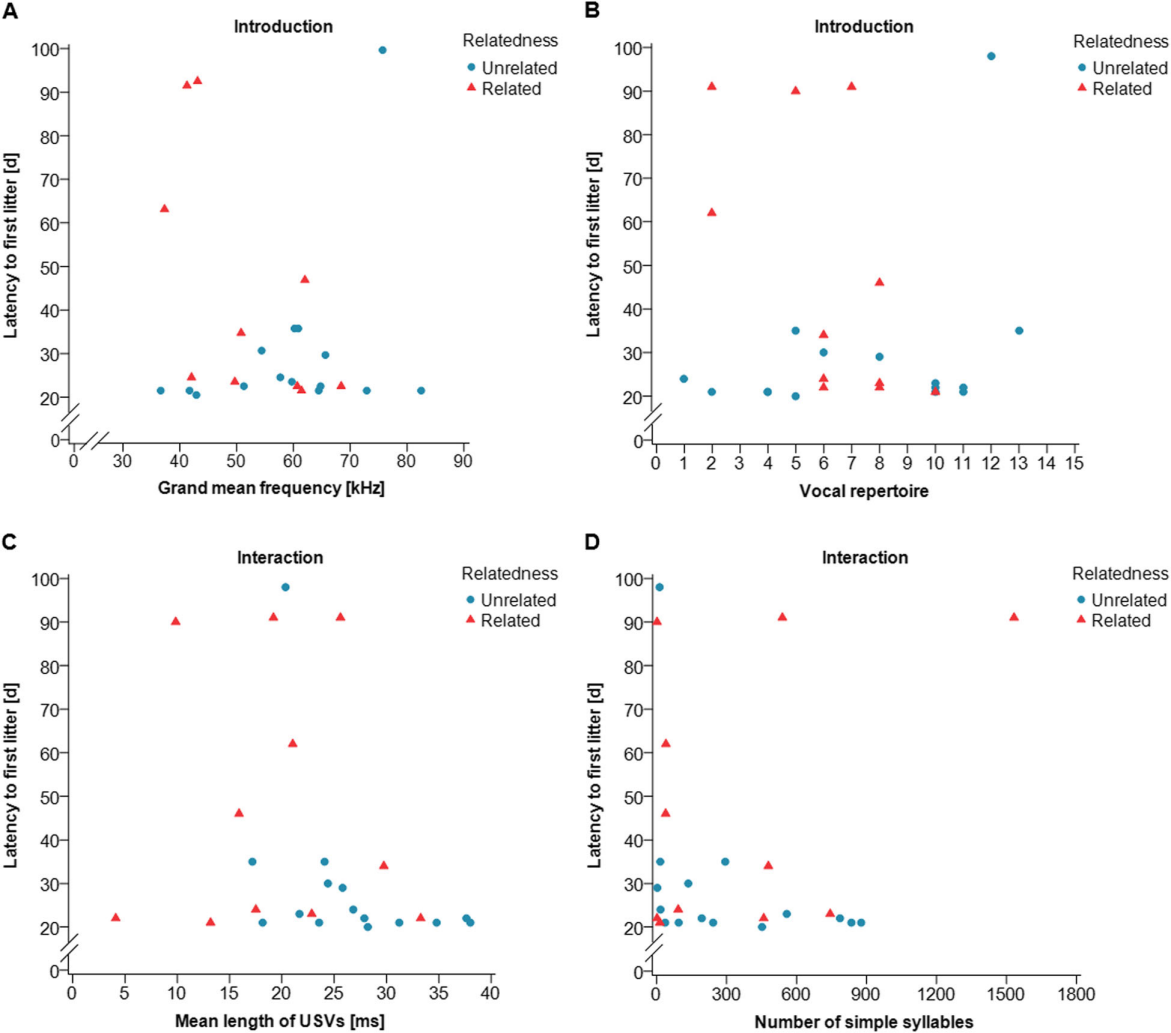
Correlation between USV emission and the latency to the first litter (LFL). Each symbol represents one breeding pair consisting of either unrelated (blue circles) or related (red triangles) individuals. During the introduction phase (**a**) the grand mean frequency of USVs (kHz) and (**b**) the vocal repertoire (number of syllable types emitted during 10 min) were correlated with LFL in related pairs. During the interaction phase (**c**) the mean length of USVs (ms) and (**d**) the number of simple syllable types (total number of simple syllables emitted during 10 min) were correlated with LFL in unrelated pairs

We tested whether USV emission correlated with the pair’s reproductive success. We found that several USV parameters correlated with the latency to the first litter. Surprisingly, we found that the results for unrelated (UR) and related (R) pairs depended upon the experimental phase. During the introduction phase, the grand mean frequency of USVs and the vocal repertoire emitted by males in related pairs was negatively correlated with the LFL (Spearman correlation: UR: *n* = 15, *r*_s_ = 0.270, *p* = 0.331, R: *n* = 10, *r*_s_ = − 0.632, *p* = 0.0498, Fig. 7a and Spearman correlation: UR: *n* = 15, *r*_s_ = 0.363, *p* = 0.184, R: *n* = 11, *r*_*s*_ = − 0.632, *p* = 0.037, Fig. 7b, respectively; see Additional file 1: Table S5). Thus, related mice emitting USVs at a higher grand mean frequency and with a larger vocal repertoire in the introduction phase had a shorter latency to the first litter. Unrelated pairs’ USV emission during the introduction phase did not correlate with LFL. During direct interactions, however, we found that the mean length of USVs negatively correlated with LFL but only in unrelated pairs (Spearman correlation: UR: *n* = 15, *r*_s_ = − 0.523, *p* = 0.046**,** R: *n* = 11, *r*_s_ = 0.123, *p* = 0.718) (Fig. 7c, Additional file 1: Table S5). Furthermore, unrelated pairs that had a higher vocal performance, tended to have a shorter LFL (Spearman correlation: UR: *n* = 15, *r*_s_ = − 0.502, *p* = 0.056**,** R: *n* = 11, *r*_s_ = 0.306, *p* = 0.360, Additional file 1: Table S5). When analyzing short, simple and complex syllable types separately, we found a significant negative correlation between the number of simple syllables and LFL (Spearman correlation: UR: *n* = 15, *r*_s_ = − 0.526, *p* = 0.040, R: *n* = 11, *r*_s_ = 0.346, *p* = 0.298) (Fig. 7d, Additional file 1: Table S5), and a trend in the correlation between the number of complex syllable types and LFL (Spearman correlation: UR: *n* = 15, *r*_s_ = − 0.472, *p* = 0.076, R: *n* = 11, *r*_s_ = 0.388, *p* = 0.238, Additional file 1: Table S5). Thus, unrelated mice emitting longer USVs and with a higher number of simple syllables during direct interactions had a shorter latency to the first litter. When using the DFA scores, which combine the USV parameters for each phase, there was no correlation between the DFA score and the latency to the first litter (Spearman correlation: introduction: *n* = 25, *r*_s_ = − 0.249, *p* = 0.230, interaction: *n* = 26, *r*_s_ = − 0.282, *p* = 0.163) (Fig. 4).

USV emission and reproductive success were not affected by male age or age differences; however, we found a negative correlation between female age and the reproductive success in unrelated but not in related pairs. Unrelated pairs with older females had a higher latency to the first litter (Spearman correlation: UR: *n* = 15, *r*_s_ = 0.826, *p* < 0.001, R: *n* = 11, *r*_s_ = 0.151, *p* = 0.658, Additional file 1: Table S6) and produced less offspring within 70 d (Spearman correlation: UR: *n* = 15, *r*_s_ = − 0.586, *p* = 0.022, R: *n* = 11, *r*_s_ = − 0.396, *p* = 0.228, Additional file 1: Table S6). Furthermore, the age of females in unrelated pairs was correlated with the vocal performance (Spearman correlation: *n* = 15, *r*_s_ = − 0.573, *p* = 0.026, Additional file 1: Table S7), vocal repertoire (Spearman correlation: *n* = 15, *r*_s_ = − 0.531, *p* = 0.042, Additional file 1: Table S7) and grand mean frequency (Spearman correlation: *n* = 15, *r*_s_ = − 0.526, *p* = 0.044, Additional file 1: Table S7) of USVs emitted during direct interactions. However, we did not find any correlation of the females’ age and USV emission in related pairs or during the introduction phase (see Additional file 1: Table S7).

**Fig. 8:**
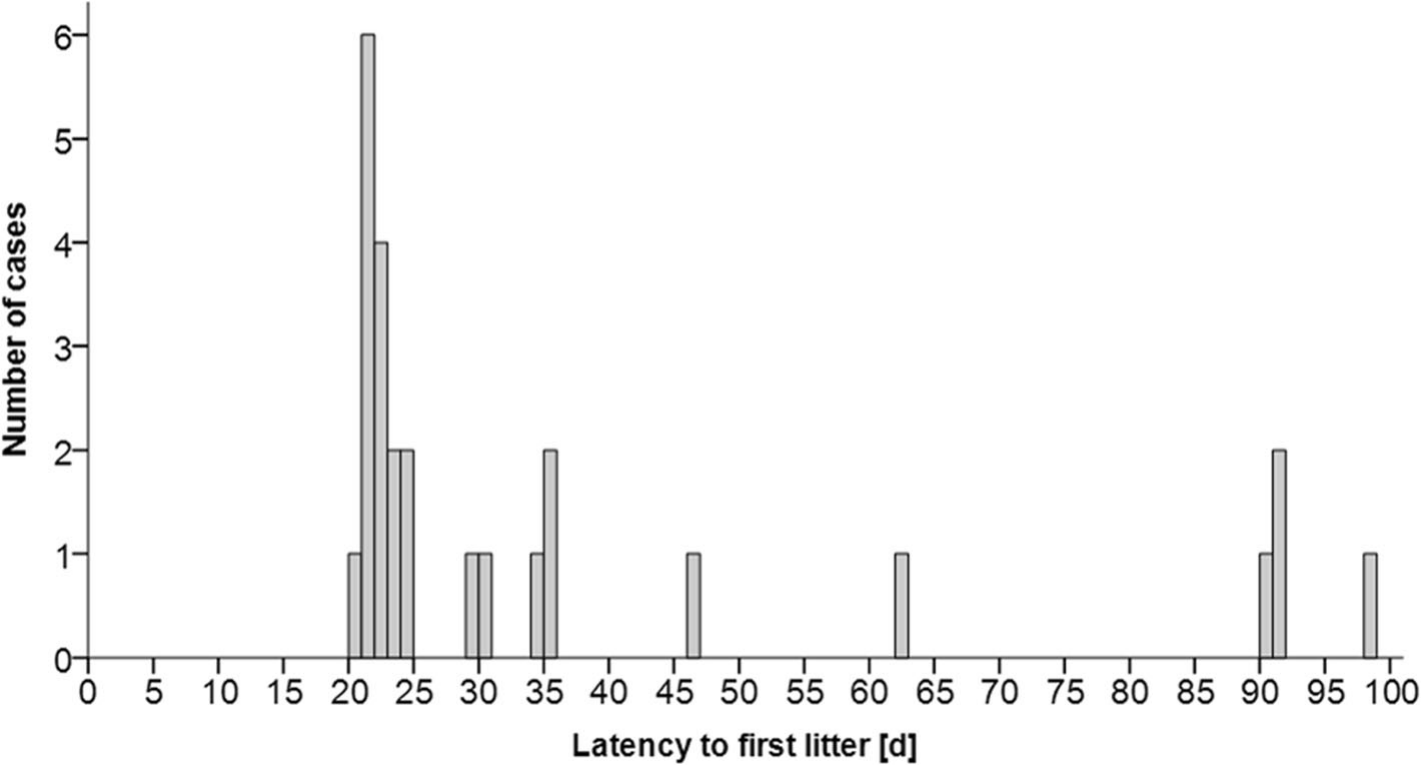
Histogram showing the frequency of the latency to the first litter. 15 pairs had a latency of <25d and 11 pairs had a latency of > 28 days. The visible cut-off at 25 days was used to divide mice into pairs with a short vs. long latency to the first litter

**Fig. 9:**
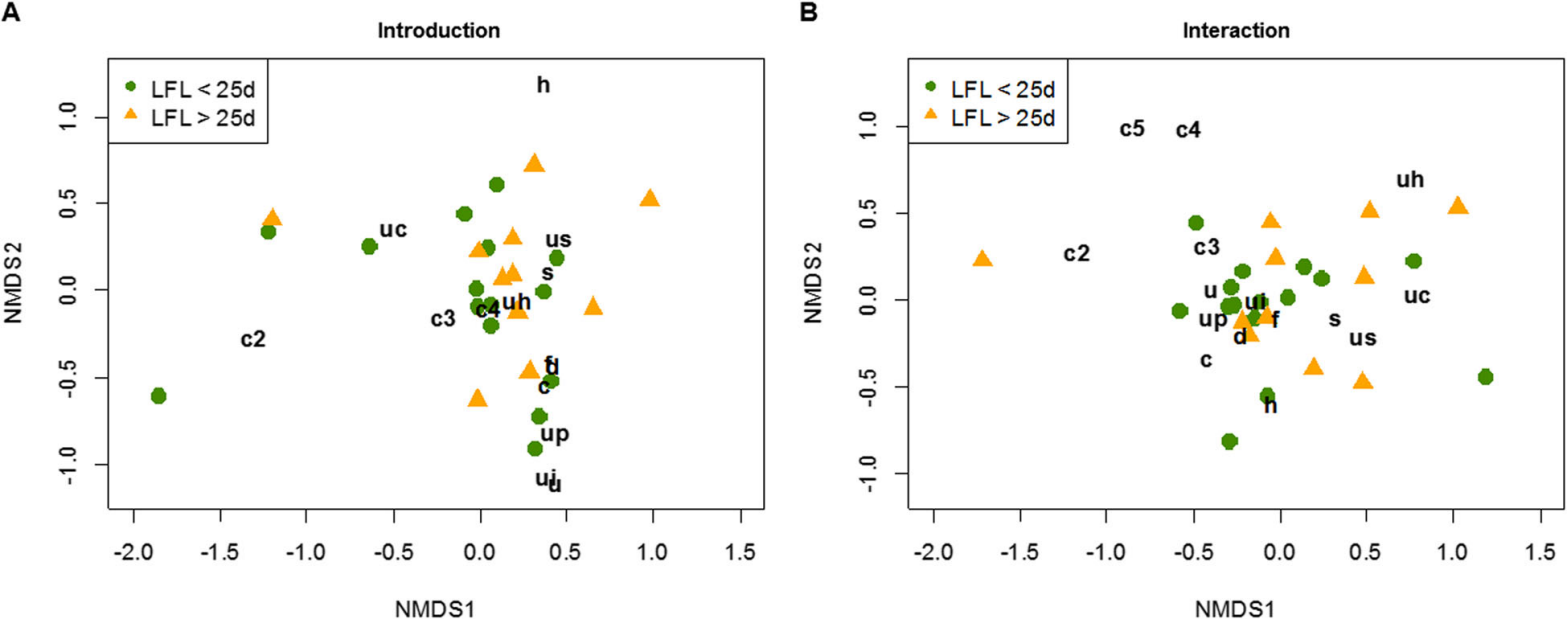
Syllable type usage depending on the latency to the first litter (LFL). NMDS plots showing syllable type usage during (**a**) introduction and (**b**) interaction phase comparing pairs with a short latency to the first litter (LFL < 25d, green circles) and pairs with a long latency to the first litter (LFL > 25d, orange triangles). Letters in black indicate the syllable types; each symbol represents one breeding pair. Distances between the symbols represent similarities of breeding pairs in the number of emitted syllable types. Short distances of symbols to letters indicate syllable types which were most representative for each breeding pair

Next, to test whether syllable type usage was associated with LFL, we divided the breeding-pairs into pairs that gave birth within 25d (short LFL) and after 25d (long LFL). We chose a cut-off at 25d for two reasons. First, visual inspection of the data showed a skewed distribution of LFL. Fifteen pairs had their first litter within 24d (20-24d), while 11 pairs had a latency of ≥29d (Fig. 8, see Additional file 1: Figure S2). Second, since the expected gestation period of mice is 21d and one estrous cycle lasts for approximately 4 days, mice with a latency to the first litter of <25d were expected to mate within the first estrous cycle. However, we found that syllable type usage in both phases did not differ between pairs with a short or long latency to the first litter (PERMANOVA: introduction: *n* = 26, F = 0.203, *p* = 0.997, interaction: *n* = 26, F = 0.835, *p* = 0.481) (Fig. 9).

## Discussion

This is the first study to record wild-derived mice during direct sexual interactions to our knowledge, and we tested whether the USVs of mice emitted during courtship depend upon the genetic compatibility of a potential mating partner, and whether USV emission is correlated with the pair’s subsequent reproductive success. Our main findings include the following results: (1) once males were allowed to directly interact with an unfamiliar female, we detected a significant increase in the number of USVs emitted, which shows that mice modulate their vocal performance during the early phases of courtship. (2) We detected longer and more complex USVs when males were experimentally paired with genetically unrelated compared to related females. This result provides further evidence that house mice show genetic kin discrimination, and the first evidence that male courtship USVs depend upon the relatedness of a potential mating partner. (3) We found that unrelated pairs of mice had higher reproductive success compared to incestuous pairings, which is consistent with inbreeding avoidance, though prenatal offspring mortality due to inbreeding depression cannot be ruled out. (4) We found that mean number and length of vocalizations of unrelated pairs were negatively correlated with the latency of the pairs’ first litter. This is the first study to our knowledge to show that USV emission depends upon genetic compatibility of mating partners, and the first to find a relationship between USV emission and *subsequent* reproductive success. Future studies are needed to test whether USV emission influences mating, whether mating influences USV emission, or both. Below we address our main findings in more detail.

### Dynamics of courtship USVs

Wild-derived house mice, unlike laboratory mice, rarely vocalize in laboratory conditions until they are presented with a stimulus mouse or its scent (Marconi et al. unpublished ms). Most males, but not all, begin vocalizing after presenting a mouse on the opposite side of a perforated partition, and especially if it is the opposite sex [15]. We expected males in this study to vocalize once they detected females through the partition and then to increase the amount and the types of USVs that they emit once they could contact and directly interact with the female. As expected, we found a large (5x) increase in the number of USVs emitted during the *interaction phase*, and the USVs were 1.5x longer compared to the preceding *introduction phase* (Fig. 1). The mice also produced a more diverse vocal repertoire, as they emitted more types of syllables during the interaction than the introduction phase. The high rates of USV emission during the interaction phase in our study were ca. 2x greater than in previous studies of wild-derived mice recorded while presenting males with only a female olfactory stimulus (female urine) [17, 20, 22, 23] or with a stimulus female separated with a divider [15, 24]. Our results indicate that once males are presented with a stimulus female, they modulate the rate of USV emission from low to higher rates of calling upon detecting and then directly interacting with and pursuing a potential mating partner. A previous study on laboratory mice similarly found that males emitted few vocalizations while the sexes were separated, and then produced high levels of USV emission during direct interactions [25]. The amount and types of USVs that mice emit during opposite-sex interactions are associated with mounting behavior [26] and the types of USVs emitted change over time during courtship and become longer and more complex (i.e., multiple frequency jumps and harmonic elements) at the end of courtship, and just before copulation [14]. Taken together, these results indicate that when male mice encounter an unfamiliar adult female, they begin vocalizing, and then continue to modulate the amount and types of USVs that they emit over time as they initiate courtship and attempt mating. Modulating USV emission over time during courtship could potentially influence male mating success, and how a male modulates his vocalizations might be more important than the total number of calls that he produces (e.g., producing too many or the wrong types of USVs too soon or too late might repel females). We observed chasing, nose-to-nose sniffing, and anogenital sniffing, but we did not observe any mating or mounting attempts in our study. Future studies are necessary with longer observation times (especially for wild mice) to document how USV emission changes over time from first encounter to copulation – and to determine how and why males modulate USV emission. One study found that females are more attracted to playbacks of complex USVs (containing more frequency jumps) than simple ones (without frequency jumps) [19]. Playback studies are now needed to manipulate the amount, types and order of USVs that females perceive during opposite-sex interactions over the stages of courtship, and to examine female responses to differences in the rate and other features of male USVs. Determining the function of dynamic modulation of male courtship USVs will be a challenge, especially since courtship vocalizations appear to be an interactive exchange between the sexes (duetting) [27–29].

The increased USV emission we found during direct interactions might be explained, at least in part, by vocalizations emitted from females during direct interactions. It was previously concluded that female mice do not vocalize during courtship, as the rate of USV (70 kHz) emission did not differ when males interacted with a surgically muted versus an intact, control female [30]. For this reason, studies on USV emission recorded during sexual interactions often assume that only males vocalize [14, 26]. More recently, however, female mice have been shown to vocalize during direct opposite-sex interactions [27–29]. However, females in these studies contributed only up to 18% of the total USVs emitted, and this is insufficient to explain the 5x increase during direct interactions that we detected in our study. Wild-derived female house mice have been shown to emit USVs when presented with a stimulus mouse separated by a divider [15, 24]. Here, we did not detect any instances of overlapping USVs, which can be an indicator that females rarely vocalized, unless they alternate their calls to avoid overlapping. While we can attribute most calls to the males during the introduction phase due to the covered compartment of the female, we cannot assume that the USVs recorded during direct interactions were emitted solely by males in our study. Nevertheless, it is unlikely that female vocalizations explain the large increase of vocalizations that we found during the direct interaction phase, and more importantly, this uncertainty does not change the interpretation of our main findings. Thus, future studies are needed to record the USV emission of both sexes during direct opposite-sex interactions in wild house mice.

### Female sexual receptivity

We expected males to modulate their USV emission depending on whether they are presented with a sexually receptive versus a non-receptive female. During the introduction phase, we found that males emitted USVs at significantly lower frequencies (43 vs. 61 kHz), and surprisingly they had a significantly *lower* vocal repertoire (6 vs. 9 syllable types) when presented with a sexually receptive female (proestrus or estrus) compared to an unreceptive (metestrus or diestrus) female (Fig. 2). However, during direct interactions we found no significant effect of female sexual receptivity on USV emission. A previous study that recorded the USVs of opposite-sex pairs of laboratory mice found that their USVs were also lower in frequency when females were in proestrus compared to diestrus [26]. The recordings were made during direct interactions, and it was assumed that this difference was due to males changing the frequency of their vocalizations, but female vocalizations were not controlled. In our study the female compartment was covered, so that the differences in USV emission we found during the introduction phase were only due to male vocalizations. Unlike this previous study, we found no evidence that female receptivity influenced the length of USVs emitted during direct interactions, and surprisingly, we found that female estrus had a negative effect on the vocal repertoire. Thus, the effects of female estrous status on male USV emission may depend on whether the mice are directly interacting and on the stage of courtship.

### Genetic relatedness and USV emission

We experimentally paired males with either a genetically related or an unrelated female, and we expected the mice to modulate their USV emission depending on the kinship of their potential mating partner. The males were unfamiliar with the stimulus females, as their sisters were from different litters. We found that mice emitted more vocalizations (introduction phase) and longer USVs (interaction phase) when presented with unrelated compared to related females. While we found that more simple syllables were emitted for an unrelated compared to a related female during the introduction phase, our multivariate (discriminant function) analysis indicated that during the interaction phase unrelated pairs emitted a higher number of complex USVs, whereas related pairs emitted a higher number of short USVs. Thus, our result shows that male house mice discriminated between genetically related versus unrelated stimulus females, and that they emitted more, longer and a higher number of complex USVs for unrelated compared to related females, indicating that males can assess the relatedness of potential mating partners, even if unfamiliar (genetic kin recognition) (reviewed in [31]). The recognition mechanisms involved here are still unclear, but mice can discriminate kinship through odor cues [32]. If USV emission provides an index of a male’s sexual arousal, as often proposed [13], then our findings suggest that male mice show mating preferences for non-kin over kin potentially to avoid inbreeding. Females have been shown to recognize siblings by their USVs, as they are more attracted to playbacks of USVs from non-siblings than siblings [17], however, we cannot rule out the possibility that females might be more attracted to unrelated males due to odor or their enhanced sexual arousal. Taken together, our findings provide further evidence for genetic kin recognition in house mice, and though USVs might mediate inbreeding avoidance [17], more playback experiments are needed to study female preferences and the recognition mechanisms. We did not compare the different degrees of relatedness (siblings vs. first cousins) due to inadequate sample sizes, and future studies are needed to investigate this question.

### Genetic relatedness and reproductive success

As expected, unrelated pairs had higher reproductive success compared to mice that we experimentally assigned to mate with close kin. Unrelated pairs produced more litters than related pairs, though their litter sizes did not differ. This result may have been due to inbreeding avoidance, though prenatal offspring mortality due to inbreeding is a non-mutually exclusive explanation for such differential reproductive success. This result did not differ when using the total number of offspring sired during the entire breeding period or only offspring born within 70d. We also found a lower latency to first litter (LFL) among unrelated pairs, though this result was also influenced by the receptivity of the female at pairing. The difference in LFL between unrelated versus related pairs was only observed when the female was initially sexually receptive. In wild-derived inbred strains of mice, it has been shown that females derived from *Mus musculus musculus* (PWD/PhJ) show a strong assortative choice when they are in estrous but not in diestrous when they could choose between *M. m. musculus* (PWK/PhJ) or *M. m. domesticus* (C57BL/6 J) males [33]. Thus, our results could be explained by females that were sexually receptive at pairing showing a greater attraction toward unrelated than related males, whereas the subsequent timing of reproduction must be explained by other factors. Our results show that unrelated pairs reproduced with a shorter latency (especially when the female was receptive) and at a faster rate (more litters) compared to related pairs.

### USV emission and reproductive success

We expected that the USV emission would predict a pair’s subsequent reproductive success (i.e., reduced latency to the first litter (LFL), increased offspring number, or both). The results supported our prediction, but surprisingly, we found different results depending upon the genetic relatedness of the pairs and the recording phase. We found significant results only during the introduction phase for the related mice and only during the interaction phase for unrelated mice. Among the genetically related pairs, we found a significant correlation between male USV emission and LFL: the males that emitted USVs at higher frequencies and with a larger vocal repertoire had a shorter latency to the first litter. This result was found during the introduction phase (which is why we can attribute the effect to the male vocalizations), but not the interaction phase. Our previous study found that mice emit USVs at higher frequencies during opposite- compared to same-sex interactions [15]. The potential functions of USVs emitted at different frequencies is not known, however, one possible explanation might be that USV frequency is related to sexual contact. Thus, if higher frequencies indicate sexual arousal, then this could explain the association with faster reproduction in the present study, however studies are needed to test for this effect. Similarly, the negative correlation between the vocal repertoire and the LFL might indicate that emitting a larger number of different syllable types can signal a higher sexual arousal of males, or might be perceived as more attractive by the female partner. Among unrelated pairs, USV emission was also correlated with LFL, but only during the interaction phase. We found a shorter latency to the first litter when unrelated males emitted longer USVs and a higher number of simple USVs during the direct interactions. We also found a negative correlation between complex USVs and LFL, though this trend was not significant. This latter result is consistent with a study in laboratory mice that found that the number of long USVs with multiple frequency jumps and harmonic USVs increase over time during courtship and mounting [14]. Additionally, this previous study found that the distribution of the duration of syllables emitted during the early phase of an interaction was different in pairs that only showed sniffing behavior compared to pairs that also showed mounting behavior. Mice exhibiting both sniffing and mounting seemed to emit longer USVs than mice that did not show mounting behavior [14]. Thus, emission of long USVs and more complex USVs might be an indicator of a higher male’s sexual arousal, and might facilitate mating. Complex and long USVs might be used by males to signal their sexual motivation [14], and simultaneously provide information about their genetic relatedness, which then might increase female receptivity [18]. Furthermore, we found that USV emission depended upon female age (among unrelated pairs), suggesting that USV emission might signal male sexual motivation. As fertility decreases with age [34], males are expected to prefer younger than older females, and here we found that unrelated pairs reproduced faster and with a larger number of offspring when the female was younger.

Finally, since our results are based on correlational evidence we cannot conclude any causal links and further studies are needed to experimentally test the effect of USV emission on mating behavior and reproductive success. Our results could be due to male preferences, female preferences, or both. Male USV emission appears to signal sexual arousal [13], and males might be more attracted to unrelated than related females. Females might be more attracted to these calls and mate faster with males that are more sexually aroused. Alternatively, females might discriminate individual males or kin versus nonkin using male USV emission [35], and might mate faster with unrelated than related males [17]. Additionally, dynamic interactions between males and females can influence the partner’s behavior, and therefore female and male preferences might not be independent from each other.

## Conclusions

In summary, our study provides evidence for dynamic modulation of courtship USVs, genetic kin recognition, and that the courtship USVs of male mice predicts their subsequent reproductive success. Our results can be useful for future breeding regimes, as USV emission could be used to screen breeding pairs during their first contact to anticipate their subsequent latency to reproduce and reproductive success. Since wild mice often show a long latency to reproduce or do not reproduce at all, this could save time and resources in the laboratory, especially when working with wild mice. Future studies are needed to manipulate the USV emission to experimentally test the effect of USV emission on mating and reproductive success. Furthermore, it is possible that USVs might even have a larger effect on male reproductive success in more natural conditions. Mice move around during courtship over a much larger area than small cages, and if male USVs help coordinate mating by keeping females nearby [36], then studies are also needed in larger areas.

## Methods

### Subjects and housing

We used wild-derived (F3) house mice (*Mus musculus musculus*). Wild mice were trapped at the Konrad Lorenz Institute of Ethology, Vienna, Austria (48°12′38″N, 16°16′54″E) in 2012 and maintained as breeding stock [for more details see 15]. We used wild-derived mice to control for age and rearing conditions. Mice were weaned at 21d and kept in mixed-sex groups with ≤4 siblings per cage until the age of 5 weeks (35d). After this time, adult males were housed individually to prevent fighting and females were housed in sister-pairs whenever possible. Mice were housed in standard Type IIL cages (36.5 × 20 × 14 cm cages, Tecniplast, Germany), with food (rodent diet 1324, Altromin, Germany) and water provided *ad libitum.* Cages were covered with stainless-steel covers (1 cm mesh width) and provided with bedding (ABEDD, Austria) and nesting material (Nestlet, Ehret, Austria). A nest box (Tecniplast, Germany) and a cardboard paper roll were provided for environmental enrichment. Home cages were kept at standard conditions (mean ± SD room temperature: 22 ± 2 °C) under a 12:12 h light-red light cycle (red lights on at 15:00). We used 26 males and 26 females, which were 249 ± 36 d old (mean ± SD) and sexually naïve at the beginning of the experiment.

### Breeding pairs and their reproductive success (RS)

Using our colony pedigree, we assigned individual males and females to two types of experimental breeding pairs: (1) 15 unrelated pairs (UR) and (2) 11 related pairs (R) with an average coefficient of relatedness (CoR) of 0.29 ± 0.2 (mean ± SD). This group included 5 pairs of siblings from different litters (CoR = 0.5) and 6 pairs of cousins that shared either two grandparents (1st degree cousins, CoR = 0.125, *n* = 3), four grandparents (double 1st degree cousins, CoR = 0.25, *n* = 1) or two great-grandparents (2nd degree cousins CoR = 0.03125, *n* = 2). Differences in sample sizes and degree of relatedness were due to constrains on the number of individuals in our colony. The age difference between males and females of the breeding pairs was 30 ± 28d (mean ± SD; median = 21d), and was not significantly different between unrelated (32 ± 30d, median = 21d) and related (28 ± 27d, median = 21d) pairs (Mann-Whitney U test: *n* = 26, Z = − 0.286, *p* = 0.775). Furthermore there was no difference of male or female age between unrelated and related pairs (Mann-Whitney U test: male age: *n* = 26, Z = − 1.272, *p* = 0.203; female age: *n* = 26, Z = − 1.326, *p* = 0.185). Breeding pairs were housed in the males’ home cage after conducting the USV recordings and under the same housing conditions described above. After 21d, pairs were checked daily for litters, and each pair’s reproductive success was documented using birth dates, litter sizes, number of litters and days that mice were kept in pairs. For further analyses of the reproductive success we used the following parameters: latency (in days) to the first litter (LFL), number of offspring in the first litter, total number of litters, total number of offspring and number of offspring/litter. The LFL of mice that failed to reproduce (*n* = 4 pairs) was defined as the number of days mice were kept in breeding pairs plus 21d (gestation time). Since mice were also bred for maintenance of our general colony, the time mice were kept in breeding pairs differed between pairs (mean ± SD: 9 ± 2 wks, range: 7–13 wks). Nevertheless, the number of days that mice were kept as breeding pairs was not significantly different between unrelated and related pairs (Mann-Whitney U test: *n* = 26, Z = − 0.523 *p* = 0.610). The minimum time pairs were kept together was 48d. To compare and standardize the breeding opportunities for all pairs, we further analyzed the parameters using only offspring born until day 70 (48d in breeding pairs +22d gestation period). Accordingly, we adjusted the number of offspring, number of litters and number of offspring/litter for pairs that reproduced also after 70d (*n* = 3 unrelated pairs). Since all pairs that reproduced delivered their first litter within 70d, the LFL only needed to be reduced to 70d for pairs that did not reproduce (*n* = 4). The analyses using the restricted dataset, did not change our main results (see Table 1), and therefore, we only present results of the full dataset, unless stated otherwise. After the breeding was terminated, all parental mice (males and females) were housed individually under standard housing conditions.

### Female estrous state

Estrous state was checked using vaginal smears 3 to 5 h prior to USV recordings and staged according to the presence or absence of vaginal cell types (light microscope with 200x magnification using a 20x objective and 10x ocular): diestrus (mainly leukocytes), proestrus (mainly nucleated epithelial cells), estrus (mainly cornified cells) and metestrus (equal combination of all three cell types) [37]. For further analyses, we pooled females in proestrus and estrus as “sexually receptive” (indicated by the absence of leukocytes) and females in metestrus and diestrus as “sexually unreceptive” (indicated by the presence of leukocytes) [33]. We used this classification since we were interested in assessing sexual receptivity rather than a particular estrous state.

### Recording apparatus and procedure

USV recordings were conducted during the mice active period under red light, i.e. after the onset of the dark phase (15:00–18:00 h) in a separate, closed room. Mice were recorded in a plexiglass cage (modified from a Type III cage, Tecniplast, Germany; floor measurements: 36.5 × 21 × 15 cm, top measurements: 42.5 × 27 × 15 cm) equally divided into two compartments by a perforated plexiglass divider (described in [15]). A clean recording cage was used for each breeding pair. Both compartments were provided with equal amount of soiled bedding from the male’s home cage. Before each recording, a male mouse was gently transferred into one of the two compartments, which was covered with a standard cage cover (1 cm mesh width). A female was then transferred into the other compartment of the cage, which allowed both olfactory and visual cues through the perforated divider, but restricted physical contact. Recordings were conducted in two consecutive phases, lasting 10 min each. In phase 1 (*introduction phase*), we aimed to record only male vocalizations, while exposed to the female on the other side of the divider, i.e. with visual, and chemical communication. To ensure that we only recorded male USVs, the female’s compartment was additionally covered with a 0.5 cm plexiglass cover, which prevented recording USVs from the female compartment (see [15]). An ultrasound microphone (USG Electret Ultrasound Microphone, Avisoft Bioacoustics / Knowles FG) was placed in a fixed position 10 cm above the center of the male compartment. For phase 2 (*interaction phase*), we removed the divider at the end of phase 1 to allow direct, physical interactions. We also exchanged the plexiglass cover with a stainless-steel cage cover, and placed the microphone 10 cm above the middle of the entire cage to ensure that USVs would be recorded from all positions in the cage. The microphone was connected to an A/D-converter (UltraSoundGate 416Hb, Avisoft Bioacoustics). Recordings were conducted on a computer (Lenovo T540p, Windows 7) using the RECORDER USGH software (Avisoft-RECORDER Version 4.2) with a sampling rate of 300 kHz and 16 bit format. During USV recordings, we videotaped the mice using an IP-camera (D-Link DCS-3710) and open source software (iSpy - Video Surveillance Software), which allowed us to observe the behavior of the mice from another room. We did not observe any mating or mating attempts (i.e. mounting, intromission, copulation) during the 10 min recordings of direct interactions. Wild-derived mice typically show a long latency to mate (compared to laboratory strains, which are selected for fast reproduction), and we never observed any mating events during such brief interactions. After the end of phase 2, both mice were gently removed from the recording cage using plastic cylinders and the male bedding was returned to the male’s home cage. Both mice were placed together into the male’s home cage to allow breeding.

### Processing and analyzing vocalizations

Sound files were processed semi-automatically in STx (S_TOOLS-ST^x^ Version 4.3.8 (9374), Acoustics Research Institute, Vienna, Austria). USVs were automatically detected using the Automatic Mouse Ultrasound Detector (A-MUD, version 3.1 [38]) and we set the threshold for element duration at 5 ms (rather than 10 ms) to increase the sensitivity in detecting ultrashort and faint elements. This threshold reduces false negatives, but increases the risk of false positive detections. We visually inspected all sound files and removed false positive and retained false negative segments. We also adjusted the length (start and end time) of the detected segments when necessary. This semi-automatic method ensured that we would include all USVs and exclude false positive segments from our analysis. The USVs were manually classified into one of 15 categories (adapted from [20, 23, 26, 39, 40]) depending upon their frequency, length and frequency modulation (Table 2). Ambiguous syllables or other sounds were verified by listening to the sounds (slowed down 15- to 20-fold). Additionally, syllables types were grouped into 3 different classes (“short syllables”, “simple syllables” and “complex syllables”, see Table 2), to reduce the number of variables in some analysis. Spectrograms for visual inspection were created using the transcription function in STx, which enabled us to scroll through the spectrogram in 2 s steps. Spectrograms were generated with a range of 50 dB (floor at -80 dB to obtain a comparable representation for all recordings), a frame of 4 ms and an overlap of 75%. The spectrograms were displayed in a Hanning window showing frequencies between 0 and 150 kHz. After classification, we ran the function *compute/update segment info* in A-MUD to compute spectrographic parameters of each detected element, including time and frequency parameters (start time, duration, mean frequency, minimum frequency and maximum frequency of each element). For one related pair, during the introduction phase only 2 USVs were emitted, which had low amplitude and the program was unable to detect frequency parameters. Thus, this pair was not included in statistical analyses when using frequency parameters of the introduction phase. All parameters were exported into an Excel-file (Microsoft) using the export-function of A-MUD, and processed for further analysis.

**Table 2:** Classification of the 15 different syllables types and grouping into 3 different syllable classes used in this study. Ambiguous syllables or other sounds were verified by acoustical inspection

Syllable shape	Syllable label	Syllable type	Syllable class	Definition	References
**(< 5 ms)**	**us**	ultrashort	Short syllables	Syllables < 91 kHz that are < 5 ms regardless of the shape	[39]
**(< 10 ms)**	**s**	short		Syllables < 91 kHz that are < 10 ms regardless of the shape	[26]
	**f**	flat	Simple syllables	Syllables < 91 kHz with < 5 kHz frequency modulation	[26]
	**d**	down		Syllables < 91 kHz that decreases in frequency for > 5 kHz	[26]
	**up**	up		Syllables < 91 kHz that increase in frequency for > 5 kHz	[26]
	**u**	u-shaped		Syllables < 91 kHz that first decrease, and then increase in frequency for > 5 kHz each	[26]
	**ui**	u-shaped inverted		Syllables < 91 kHz that first increase, then decrease in frequency for > 5 kHz each	[26]
	**c**	complex	Complex syllables	Syllables < 91 kHz that contain ≥2 directional changes in frequency and > 5 kHz modulation of frequency	[26]
	**c2**	complex 2		Syllables < 91 kHz consisting of 2 elements separated by 1 frequency-jump without time separation	[20]
	**c3**	complex 3		Syllables < 91 kHz consisting of 3 elements separated by 2 frequency-jumps without time separation	[20]
	**c4**	complex 4		Syllables < 91 kHz consisting of 4 elements separated by 3 frequency-jumps without time separation	Added category for our classification
	**c5**	complex 5		Syllables < 91 kHz consisting of ≥5 elements separated by ≥4 frequency jumps without time separation	Added category for our classification
	**h**	harmonic		Syllables < 91 kHz that have an harmonic element	[26]
**(> 91 kHz)**	**uh**	ultra high		All syllables > 91 kHz regardless of the shape	[23]
	**uc**	unclassified		Syllables that do not fit any other of the 14 categories due to background noise or that lack clearly defined spectrographic features (shape)	[40, 41]

### Statistical analyses

To quantify the total USV emission rate, we used the total number of USVs (*vocal performance*) recorded in each 10 min phase. To quantify the usage of different syllable types, we used the total number of USVs of each syllable type emitted per 10 min phase. The amount of different syllable types used by each pair is defined as their *vocal repertoire* (0–15 different syllable types). For quantifying additional spectrographic parameters (time and frequency parameters), we calculated means (e.g., mean length) or grand means (e.g., grand mean frequency) for each pair, separately for each parameter and for each 10 min recording. The mean length was calculated from the length of each USV averaged over all USVs in each recording. To calculate the grand mean frequency we used the mean frequency of each USV (i.e. the average frequency of the frequency track (contour) measured by AMUD) and calculated the average over all USVs in each recording [15]. We examined data distributions and homogeneity of variances using the Kolmogorov–Smirnov test and Levene’s test, respectively, and we used non-parametric statistical tests if the assumptions for parametric statistics were not met. We tested for normal distribution separately for each phase and the different groups depending on the question (introduction vs interaction, receptive vs unreceptive, related vs. unrelated). If possible, we transformed the data to reach normal distribution. USV count data were square-root transformed, after adding 0.5 to the data (sqrt(x + 0.5)), and LFL was log-transformed (log(x)). Sqrt transformation of the USV count data resulted in normal distribution when comparing related vs unrelated pairs. Log-transformation of LFL was used to test for interactions between relatedness and receptivity. Detailed variable definitions and raw data which were used for statistical analyses are provided as additional file (Additional file 2). We used two-tailed tests, and results were considered statistically significant at α ≤ 0.05 and presented as mean ± SD, unless stated otherwise. Statistical tests were conducted using SPSS (IBM SPSS Statistics 24) and RStudio (R-Version 3.5.1 [42], using the functions “vegdist”, “anosim”, “adonis2” and “metaMDS” included in the package “vegan” [43]).

Whenever we tested for interactions between the female’s sexual receptivity and relatedness to the male, we conducted a generalized linear model (GZLM) including the pairs’ relatedness, the females’ receptivity and the interaction of both as fixed factors. Additionally, we performed different multivariate methods to investigate whether different USV parameters and the syllable type usage depend upon the relatedness of the pairs. We conducted a discriminant function analysis (DFA) to test whether mice could be classified into unrelated or related pairs and which USV features had the main effect in discriminating between the two groups. We conducted DFA separately for the introduction and interaction phase, and included the following features: mean USV length, grand mean USV frequency, vocal repertoire, number of short syllables, number of simple syllables and number of complex syllables. These parameters were included in the DFA in order to combine spectrotemporal features with parameters of syllable diversity and complexity. Since we only compared two groups (unrelated vs. related pairs), the DFA resulted in only one discriminant function axis. For visual representation of the results, we plotted the DFA score against the latency to first litter as a variable describing the reproductive success, as this was one of our main study questions. To describe differences in syllable type usage between unrelated and related pairs, we conducted PERMANOVA (permutational multivariate analysis of variance) on the number of USVs emitted within each of the 15 syllable types. PERMANOVA is a non-parametric alternative to other multivariate statistics (such as MANOVA), which works on permutations of a dissimilarity measure [44, 45]. We conducted the analysis with 999 permutations by running the function “adonis2” in the R-Package “Vegan” [43], using the rank based Bray–Curtis dissimilarity indices. Non-metric multidimensional scaling (nMDS) plots were created for visual representation of the results. The stress value of the plots describes whether the 2-dimensional nMDS plot sufficiently summarizes the relationship of the multidimensional data [46]. Stress values < 0.05, < 0.1 and < 0.2 will give an excellent, good or intermediate representation of the data, respectively. Results with stress values of 0.2–0.3 should be interpreted carefully, while stress values > 0.3 indicate arbitrary representation of the data in the 2-dimensional space [46]. To examine the relationship between USV emission and reproductive success, we conducted Spearman rank correlations separately for each phase and for unrelated and related pairs.

## Supplementary information

**Additional file 1.** Supplementary material, containing additional figures (Figures S1 and S2) and summary tables of statistical results (Tables S1 – S7).

**Additional file 2.** Original dataset containing the raw data and variable definitions used in this article.

## Data Availability

All data generated or analyzed during this study are included in this published article and its supplementary information files (Additional file 2).
