# Supplementary material for: Ultrasonic vocalizations in house mice depend upon genetic relatedness of mating partners and correlate with subsequent reproductive success
Source: Front Zool. 2020 Apr 3;17:10. doi: 10.1186/s12983-020-00353-1 (PMC7118824; doi:10.1186/s12983-020-00353-1)
Supplement: Supplementary file 1 — Additional file 1. Supplementary material, containing additional figures (Figures S1 and S2) and summary tables of statistical results (Tables S1 – S7). [file 12983_2020_353_MOESM1_ESM.docx]

**Supplementary material**


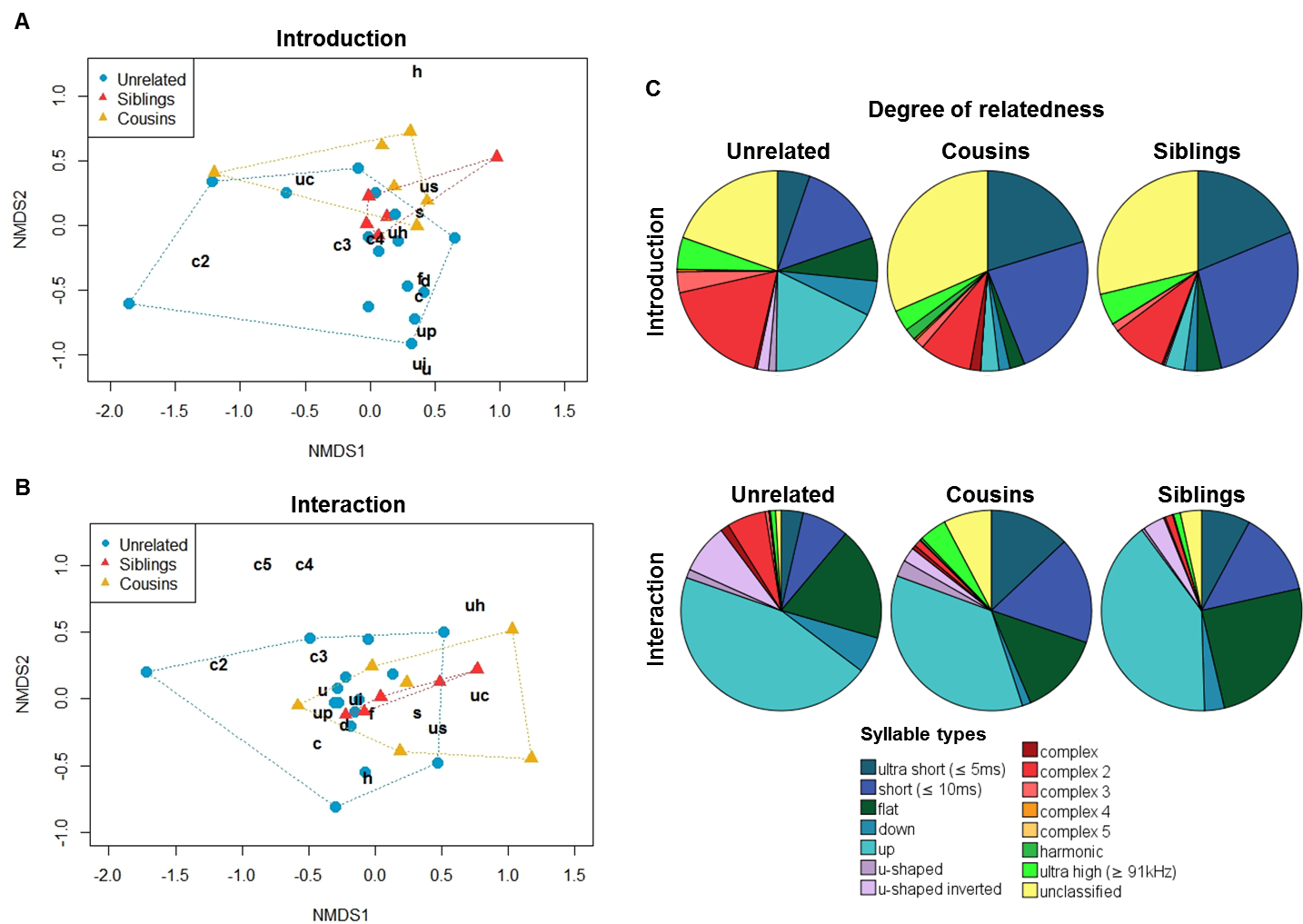


**Figure S1: Syllable type usage presented in non-metric multidimensional scaling (nMDS) plots and pie charts.** NMDS plots of syllable types emitted during **(A)** introduction and **(B)** interaction phase comparing pairs consisting of unrelated mice (blue dots, n=15), cousins (orange triangles, n=6) and siblings (red triangles, n=5). Letters in black indicate the syllable types and each symbol represents one breeding pair. Distances between the symbols represent similarities of breeding pairs in the syllable type usage. Short distances of symbols to letters indicate syllable types that were most representative for each breeding pair. **(C)** Pie charts representing proportions of each syllable type used by pairs consisting of unrelated mice, cousins and siblings (horizontal) during introduction and interaction phase (vertical). Both visualizations show similar results of syllable type usage.


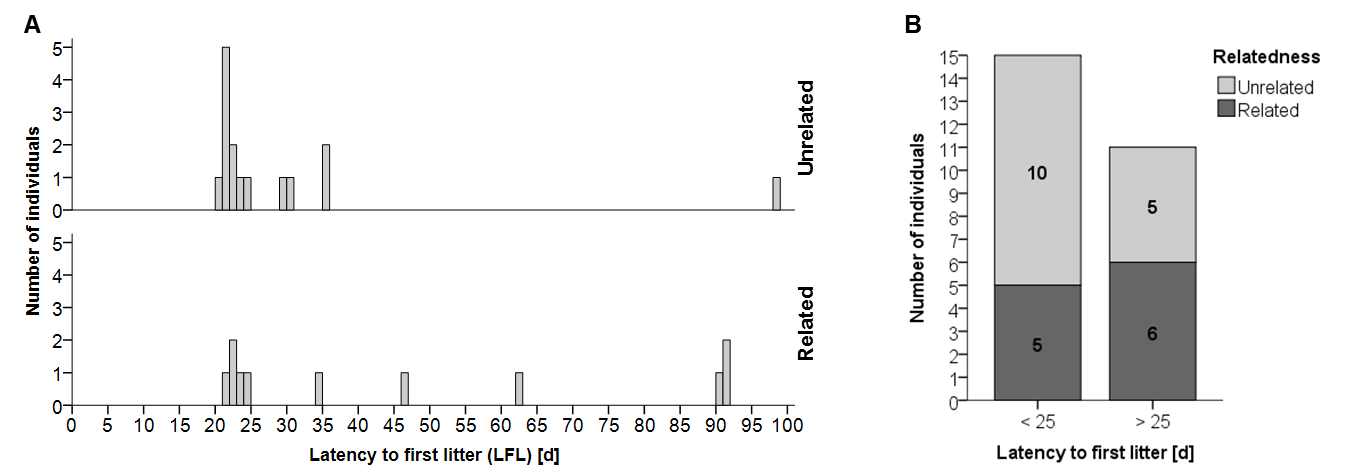


**Figure S2:** **Latency to the first litter (LFL) of unrelated and related pairs.** **(A)** Distribution of LFL separately for unrelated (upper panel) and related (lower panel) pairs. **(B)** Number of unrelated (light gray) and related (dark gray) breeding pairs with a short (<25d) or long (>25d) latency to the first litter (LFL).

**Table S1: Comparison of USV parameters measured during introduction and interaction phases.** Significant results with p < 0.05 are shown in bold.

| **USV parameter** | **Wilcoxon signed-rank test** | | |
| --- | --- | --- | --- |
|  | **Z** | **p-value** | **n** |
| Mean USV length | **-3.467** | **0.0005** | **26** |
| Grand mean USV frequency | -1.466 | 0.143 | 26 |
| Vocal performance | **-3.264** | **0.001** | **26** |
| Vocal repertoire | **-3.912** | **0.00009** | **26** |
| Number of short USVs | -1.815 | 0.069 | 26 |
| Number of simple USVs | **-3.565** | **0.0004** | **26** |
| Number of complex USVs | -1.037 | 0.300 | 26 |

**Table S2: Comparison of USV parameters measured in the presence of receptive or unreceptive females.** Results are shown separately for the introduction and interaction phases. Significant results with p < 0.05 are shown in bold.

| **USV parameter** | **Mann-Whitney U test** | | | | | |
| --- | --- | --- | --- | --- | --- | --- |
|  | **Introduction** | | | **Interaction** | | |
|  | **Z** | **p-value** | **n** | **Z** | **p-value** | **n** |
| Mean USV length | -1.234 | 0.217 | 26 | -0.309 | 0.758 | 26 |
| Grand mean USV frequency | **-2.502** | **0.012** | **25** | -0.463 | 0.643 | 26 |
| Vocal performance | -1.313 | 0.189 | 26 | -1.698 | 0.090 | 26 |
| Vocal repertoire | **-2.434** | **0.015** | **26** | -1.643 | 0.100 | 26 |
| Number of short USVs | -1.340 | 0.180 | 26 | -1.930 | 0.054 | 26 |
| Number of simple USVs | **-2.506** | **0.012** | **26** | -1.569 | 0.117 | 26 |
| Number of complex USVs | -1.634 | 0.102 | 26 | -1.549 | 0.121 | 26 |

**Table S3: Comparison of USV parameters measured in the presence of related or unrelated females.** Results are shown separately for the introduction and interaction phase. T-tests/Welch’s t-tests were conducted for normal distributed parameters, Mann-Whitney U tests for non-normal distributed data. Square root-transformations (sqrt(x+0.5)) normalized some of the parameters. We also report results of some transformed and untransformed parameters, to keep data comparable with the other results. Significant results with p < 0.05 are shown in bold.

| **USV parameter** | **Unpaired t-test / unpaired Welch's t-test** | | | | | |
| --- | --- | --- | --- | --- | --- | --- |
|  | **Introduction** | | | **Interaction** | | |
|  | **t** | **p-value** | **n** | **t** | **p-value** | **n** |
| Mean USV length | **3.161** | **0.005** | **26** | **2.499** | **0.020** | **26** |
| Grand mean USV frequency | 1.580 | 0.128 | 25 | 0.933 | 0.360 | 26 |
| sqrt (Vocal performance + 0.5) | 1.963 | 0.066 | 26 | -0.038 | 0.970 | 26 |
| Vocal repertoire | 1.035 | 0.311 | 26 | 0.773 | 0.447 | 26 |
| sqrt (Number of short USVs + 0.5) | -0.069 | 0.945 | 26 | -0.699 | 0.491 | 26 |
| sqrt (Number of simple USVs + 0.5) | see Mann-Whitney U test | | | -0.005 | 0.996 | 26 |
| sqrt (Number of complex USVs +0.5) | 1.440 | 0.163 | 26 | 1.207 | 0.239 | 26 |
| **USV parameter** | **Mann-Whitney U test** | | | | | |
|  | **Introduction** | | | **Interaction** | | |
|  | **Z** | **p-value** | **n** | **Z** | **p-value** | **n** |
| Vocal performance | -0.961 | 0.336 | 26 | -0.026 | 0.979 | 26 |
| Number of simple USVs | -1.917 | 0.055 | 26 | -0.285 | 0.775 | 26 |
| sqrt (Number of simple USVs + 0.5) | -1.917 | 0.055 | 26 | see T-test | | |

**Table S4: Tests for interactions between pair relatedness and female receptivity using generalized linear models (GZLM).** Results are shown separately for the introduction and interaction phase.

| **USV parameter** | **GZLM (Relatedness * Receptivity)** | | | | | |
| --- | --- | --- | --- | --- | --- | --- |
|  | **Introduction** | | | **Interaction** | | |
|  | **Wald-Chi-Square** | **p-value** | **n** | **Wald-Chi-Square** | **p-value** | **n** |
| Mean USV length | 0.290 | 0.590 | 26 | 0.017 | 0.896 | 26 |
| Grand mean USV frequency | 0.404 | 0.525 | 26 | 1.761 | 0.185 | 26 |
| sqrt (Vocal performance + 0.5) | 0.133 | 0.715 | 26 | 0.746 | 0.388 | 26 |
| Vocal repertoire | 0.006 | 0.937 | 26 | 0.446 | 0.504 | 26 |

**Table S5:** **Relationship** **between USV parameters and the latency to the first litter (LFL).** Results of Spearman rank correlations are shown separately for the introduction and interaction phase and for unrelated and related pairs. Significant results with p < 0.05 are shown in bold.

| **Spearman rank correlations** | | | | | | | |
| --- | --- | --- | --- | --- | --- | --- | --- |
| **USV parameter** | | **Latency to the first litter (LFL)** | | | | | |
|  |  | **Introduction phase** | | | **Interaction phase** | | |
|  |  | **r_s_** | **p-value** | **N** | **r_s_** | **p-value** | **N** |
| **Unrelated** | Mean USV length | -0.038 | 0.892 | 15 | **-0.523** | **0.046** | **15** |
|  | Grand mean USV frequency | 0.270 | 0.331 | 15 | -0.275 | 0.321 | 15 |
|  | Vocal performance | 0.200 | 0.475 | 15 | -0.502 | 0.056 | 15 |
|  | Vocal repertoire | 0.363 | 0.184 | 15 | -0.438 | 0.103 | 15 |
|  | Number of short USVs | 0.453 | 0.090 | 15 | -0.296 | 0.284 | 15 |
|  | Number of simple USVs | 0.218 | 0.435 | 15 | **-0.536** | **0.040** | **15** |
|  | Number of complex USVs | 0.281 | 0.311 | 15 | -0.472 | 0.076 | 15 |
| **Related** | Mean USV length | -0.315 | 0.345 | 11 | 0.123 | 0.718 | 11 |
|  | Grand mean USV frequency | **-0.632** | **0.0498** | **10** | -0.237 | 0.482 | 11 |
|  | Vocal performance | -0.442 | 0.174 | 11 | 0.306 | 0.360 | 11 |
|  | Vocal repertoire | **-0.632** | **0.037** | **11** | 0.343 | 0.302 | 11 |
|  | Number of short USVs | -0.498 | 0.119 | 11 | 0.498 | 0.119 | 11 |
|  | Number of simple USVs | -0.417 | 0.202 | 11 | 0.346 | 0.298 | 11 |
|  | Number of complex USVs | -0.332 | 0.319 | 11 | 0.388 | 0.238 | 11 |

**Table S6:** **Relationship** **between reproductive success and the female age.** Results of Spearman rank correlations are shown separately for unrelated and related pairs. Significant results with p < 0.05 are shown in bold.

| **Spearman rank correlations** | | | | |
| --- | --- | --- | --- | --- |
| **Parameters of reproductive success** | | **Female age** | | |
|  |  | **r_s_** | **p-value** | **N** |
| **Unrelated** | Latency to first litter (LFL) | **0.826** | **0.000** | **15** |
|  | N. of offspring in 70d | **-0.586** | **0.022** | **15** |
|  | N. of litters in 70d | -0.416 | 0.123 | 15 |
|  | N. of pups / litter in 70d | -0.485 | 0.067 | 15 |
|  | N. of offspring in 1. litter | -0.477 | 0.072 | 15 |
| **Related** | Latency to first litter (LFL) | 0.151 | 0.658 | 11 |
|  | N. of offspring in 70d | -0.396 | 0.228 | 11 |
|  | N. of litters in 70d | -0.213 | 0.529 | 11 |
|  | N. of pups / litter in 70d | -0.239 | 0.479 | 11 |
|  | N. of offspring in 1. litter | -0.220 | 0.515 | 11 |

**Table S7:** **Relationship between USV parameters and the female age.** Results of Spearman rank correlations are shown separately for the introduction and interaction phase and for unrelated and related pairs. Significant results with p < 0.05 are shown in bold.

| **Spearman rank correlations** | | | | | | | |
| --- | --- | --- | --- | --- | --- | --- | --- |
| **USV parameter** | | **Female age** | | | | | |
|  |  | **Introduction phase** | | | **Interaction phase** | | |
|  |  | **r_s_** | **p-value** | **N** | **r_s_** | **p-value** | **N** |
| **Unrelated** | Mean USV length | -0.045 | 0.874 | 15 | -0.472 | 0.076 | 15 |
|  | Grand mean USV frequency | 0.257 | 0.356 | 15 | **-0.526** | **0.044** | **15** |
|  | Vocal performance | 0.070 | 0.804 | 15 | **-0.573** | **0.026** | **15** |
|  | Vocal repertoire | 0.127 | 0.651 | 15 | **-0.531** | **0.042** | **15** |
| **Related** | Mean USV length | -0.009 | 0.979 | 11 | -0.355 | 0.285 | 11 |
|  | Grand mean USV frequency | -0.358 | 0.310 | 10 | 0.045 | 0.894 | 11 |
|  | Vocal performance | -0.237 | 0.483 | 11 | -0.564 | 0.071 | 11 |
|  | Vocal repertoire | -0.543 | 0.084 | 11 | -0.399 | 0.224 | 11 |
